# Beginning at the Beginning

**Published:** 1924-08

**Authors:** 


THE
### HOSPITAL and HEALTH REVIEW

### BEGINNING AT THE BEGINNING.

Its youth notwithstanding, the English-speaking Conference on Infant Welfare is rapidly becoming an organisation of real importance. During the past month it has held a Conference which, although it was only the third of its kind, was attended by seven hundred delegates, some of whom came from the Dominions and some from America. As will be seen from the account of the proceedings, which we print on another page, the Conference discussed many matters of moment connected with the saving of infant life and the care of mothers, and Mr. Wheatley, the Minister of Health, delivered an address which emphasised the urgency of the whole matter. Almost simultaneously Mr. Benjamin Broadbent contributed to the *Times* two articles on the Saving of Infant Life which seem likely to mark a new point of departure in a subject which is rapidly focussing the attention of great numbers of people whose interest in it has hitherto been chiefly passive. The truth is that the country is only now beginning to awaken to the fact, long ago clearly apprehended by those closely or professionally concerned, that it is hopeless to expect a really satisfactory level of public health unless we care for that health from the beginning, and watch over the child not merely from the hour of its birth, but long before its birth. Infant Welfare starts with Maternal Welfare; the two things are intimately associated, and all these Conferences and Baby Weeks and other methods of propaganda would be of little value did they not fully recognise this interrelation. At present, however, it would seem that we are more successful in preserving infant life than in saving maternal life. In less than a quarter of a century we have reduced infant mortality by more than one-half, yet the ratio of maternal deaths to live births is still pretty much what it was when the century began. The explanation would seem to be that we have supervised the children much more vigorously and systematically than their mothers. In this country four mothers die to every thousand live births, which is exactly double the American figure, as given by one of the delegates from the United States. This is distinctly humiliating for a country which has rightly regarded itself as being the inventor of sanitary science and the leader in all movements for the improvement of the public health.

There is thus abundant scope for educative measures and for initiative and energy in their application. It is an odd circumstance that, although bodily health is the primary asset of every man and woman, a large proportion of them take little care of it until care is more or less forced upon them. The circumstances of many mothers make such care difficult; it is the business of the State to see that it is made easier. In the meantime Mr. Broadbent has been telling a remarkable story of what can be accomplished in the salving of infant life by careful supervision and by the co-ordination of effort between official and voluntary agencies —a story which is full of encouragement. If we can save the children we can save their mothers. Mr. Broadbent set to work to discover the best and the worst towns in which a child can be born. He tells us the ten best and enlarges upon those that stand at the head of the ten. About the worst he keeps silence save to remind us that their average infant death-rate over ten years is virtually double that of the towns at the other end of the scale. To the general astonishment Oxford comes first with 64 deaths per 1,000 births ; Cambridge is a good second with 65 ; Worcester and Swindon follow with 72 and 75 respectively. It would be unwise to jump to the conclusion that the climate of low-lying, damp, foggy places like Oxford and Cambridge is especially favourable to infant life. It is true they are both very well scavenged, which counts for much. But the real reason for the low rates of the premiated towns —always, be it understood, on a decennial average— is the especial care they take of infant life. These happy results flow from the close co-ordination of the municipal medical service with voluntary effort. The Medical Officer, perhaps an assistant special Medical Officer, the Health Visitors, the School Medical Service are all linked up with those voluntary organisations of ladies which, in most cases, were doing splendid work long before the municipalities entered this field. There is no such organisation at Swindon, and Swindon stands fourth on the list. In each case similar machinery is at work—visiting in the homes, " talks " to mothers, the supply of dinners, milk, cod liver oil, virol, lessons in sewing, cheap body garments, weighing of the children. In Worcester 95 per cent. of the working mothers are in touch with the Infant Welfare work.

The question with which we are now faced is the best and quickest method of converting the backward towns into Oxfords, Cambridges, and Worcesters. Many of them are by no means favourably situated from the point of view of the health of the newly born. Some have rigorous climates or many slums, or are indifferently scavenged ; in a certain number these disadvantages are cumulated. The towns in which the mothers work in factories before and after the birth of their children are numerous. Not all of these drawbacks are removable, and hard conditions mean long effort and, it may be, only moderate success. But any degree of success is of definite importance, and it is obvious that, at the worst, an enormous improvement can be made in the communities which suffer from the highest infant mortality. Mr. Broadbent points to the need for further investigation into the causes which make for or against the rearing of children of tender age, and suggests an inquiry by the Carnegie Trust, the Rockefeller Foundation, the Ministry of Health, or a Royal Commission. The country is not enamoured of Royal Commissions, which have too often been the grave of reform, but no other investigating body can possess such wide powers or claim so large a share of public attention. Experience shows that save myriads of child lives, and it is not less certain we can, if we will, that we can save every year many hundreds of mothers who at present perish in the flower of their age. Something has, however, yet to be done to bring home the nation's duty to the nation's conscience, and the sooner we set to work, by Royal Commission or otherwise, upon that task the sooner will the present waste of young life be stemmed.

